# Predictive validity of the GOSLON Yardstick index in patients with unilateral cleft lip and palate: A systematic review

**DOI:** 10.1371/journal.pone.0178497

**Published:** 2017-06-01

**Authors:** Cindy Buj-Acosta, Vanessa Paredes-Gallardo, José María Montiel-Company, Alberto Albaladejo, Carlos Bellot-Arcís

**Affiliations:** 1 Orthodontics Teaching Unit, Department of Dental Medicine, Faculty of Medicine and Dentistry, University of Valencia, Valencia, Spain; 2 Preventive Teaching Unit, Department of Dental Medicine, Faculty of Medicine and Dentistry, University of Valencia, Valencia, Spain; 3 Orthodontics Department, Faculty of Dentistry, University of Salamanca, Salamanca, Spain; University of Washington, UNITED STATES

## Abstract

Among the various indices developed for measuring the results of treatment in patients born with unilateral cleft lip and palate (UCLP), the GOSLON Yardstick index is the most widely used to assess the efficacy of treatment and treatment outcomes, which in UCLP cases are closely linked to jaw growth. The aim of this study was to conduct a systematic review to validate the predictability of growth using the GOSLON Yardstick in patients born with UCLP. A systematic literature review was conducted in four Internet databases: Medline, Cochrane Library, Scopus and Embase, complemented by a manual search and a further search in the databases of the leading journals that focus on this topic. An electronic search was also conducted among grey literature. The search identified a total of 131 articles. Duplicated articles were excluded and after reading titles and abstracts, any articles not related to the research objective were excluded, leaving a total of 21 texts. After reading the complete text, only three articles fulfilled the inclusion criteria. The results showed a predictive validity of between 42.2% and 64.7%, which points to a lack of evidence in the literature for the predictive validity of the GOSLON Yardstick index used in children born with UCLP.

## Introduction

Unilateral cleft lip and palate (UCLP) is one of the most common birth defects. To correct this anomaly, patients born with UCLP need surgery and/or other complex procedures. Cleft lip or palate may constitute a single anomaly or may form part of multiple birth defects. Patients born with this condition often present a series of esthetic and functional deformities, in addition to the specific malformations deriving from UCLP by definition [1].

Complications associated with UCLP include deficient maxillary growth and a high incidence of Class III malocclusion. For this reason treatment of patients born with UCLP requires a multidisciplinary approach, starting with surgical repair of both the lip (usually performed when the baby is 3 months old) and the palate (performed at any time between the ages of 6 and 14 months). Various studies have affirmed that when this primary surgery is carried out inadequately this may compromise future facial growth, dental development [2], and speech [3].

To assess and compare the results of the early management of a child born with UCLP, it is essential to establish a reliable method of determining dental arch relationships. In the context of orthodontics, some type of clinical index or system of categorization is used to allot a classification, in the form of a numeric or alphanumeric score, to the individual patient’s occlusion [4]. In the case of children born with UCLP, specific indices are used to provide an objective measurement of the severity of malocclusion [5].

The most relevant tools used for this purpose described in the literature for measuring constriction of the upper arch in patients born with UCLP are: the GOSLON Yardstick index [2], the index for 5-year-old children [6]; and the Modified Huddart Bodenham scoring system (MHB) [7,8]. The most widely used of these clinical tools, the GOSLON (Great Ormond Street, London and Oslo) Yardstick index was developed by Mars *et al*. [2], a standardized method for categorizing treatment outcomes based on the analysis of dental relationships (anteroposterior arch, vertical labial segment and transverse relationships) using study models of children born with UCLP in late mixed dentition (10 years of age). This measurement system classifies patients as five groups (from excellent to poor) according to the prediction of clinical results of orthodontic treatment alone or in combination with orthognathic surgery (Table 1).

**Table 1:** Prediction of treatment necessary for patients in to each GOLSON Yardstick classification.

	Prediction
Group 1—excellent	Patients require either straightforward orthodontic treatment or none at all.
Group 2—good	
Group 3—fair	Patients require complex orthodontic treatment to correct the Class III malocclusion and possibly other arch malrelationships, but a good result can be anticipated.
Group 4—poor	Cases are at the limits of orthodontic treatment without orthognatic surgey to correct skeletal malrelationships, and if facial growth is unfavorable, orthognatic surgery will be required.
Group 5—very poor	Cases require orthognatic surgery to correct skeletal malrelationships if there is to be any prospect of obtaining satisfactory oclusal relationships

While a large number of inter-center studies have compared different UCLP treatment procedures [4,9–20], to date few studies have evaluated the changes produced in the GOLSON Yardstick index in the long term in patients born with UCLP. It should be stressed that a change produced in the GOLSON index applied at the age of 10 years entails for the patient and his/her family a change in prognosis, expected growth pattern, and the expectations of treatment success. For this reason, the aim of this study was to perform a systematic literature review to assess the predictive validity of the GOLSON Yardstick index.

### Methods

This systematic literature review fulfilled PRISMA statement guidelines (Preferred Reporting Items for Systematic Reviews and Meta-Analyses) [21]. The review protocol has been registered in the PROSPERO register (number CRD42016049577).

### Study selection criteria

Of all the literature reviewed, only those articles that fulfilled the following inclusion criteria were selected: meta-analyses, systematic reviews, randomized clinical trials (RCTs), case reports, case-control studies, and cohort studies. Retrospective and prospective studies published during the last 30 years (1986–2016) were included. The literature search was conducted on October 17^th^ 2016. The language the article was published in was not an exclusion criterion.

### Search strategy and article screening

A rigorous electronic search was made in the Internet databases Medline, Cochrane Library, Scopus and Embase. An electronic search among grey literature was also conducted in Open Grey and the New York Academy of Medicine Grey Literature Report. Searches were also made in the databases of The Cleft Palate-Craniofacial Journal and The European Journal of Orthodontics.

The search used combinations of the following MeSH (Medical Subject Headings) terms, as well as other non-MeSH terms that might identify articles directly related the research area under investigation: *“Cleft lip palate”*, *“Cleft lip and palate”*, *“Cleft lip repair”*, *“Cleft palate dental”*, *“Cleft lip and palate review”*, *“Cleft lip and palate orthodontics”*, *“Cleft lip and palate classification”*, *combined with the terms “GOSLON”*, *“GOSLON Yardstick”*, *“Cleft Palate” AND “GOSLON Yardstick”*, *“GOSLON” AND “reproducibility”*, *“GOSLON” AND “predictability”*, *“Cleft lip” AND “palate” OR “predictability”*. The electronic search was complemented by a manual search among bibliographic references of the articles found in the electronic search, to locate any further articles that the primary search had failed to identify. Articles investigating the predictive validity of the GOSLON Yardstick were selected.

### Data extraction and variables

The following variables were registered: author, year of publication, study type (retrospective/prospective), sample size, loss of individual subjects, demographic variables (age and sex), inclusion and exclusion criteria, follow-up periods, and the results (reproducibility and predictability, conclusions).

### Quality assessment

The researchers analyzed the quality of each study independently, using the QUADAS (quality assessment of diagnostic accuracy studies) tool [22]. When a discrepancy occurred between researchers, consensus was reached by discussion, or when this was not possible, a third researcher was consulted.

## Results

### Article selection and flow diagram

**Fig 1:**
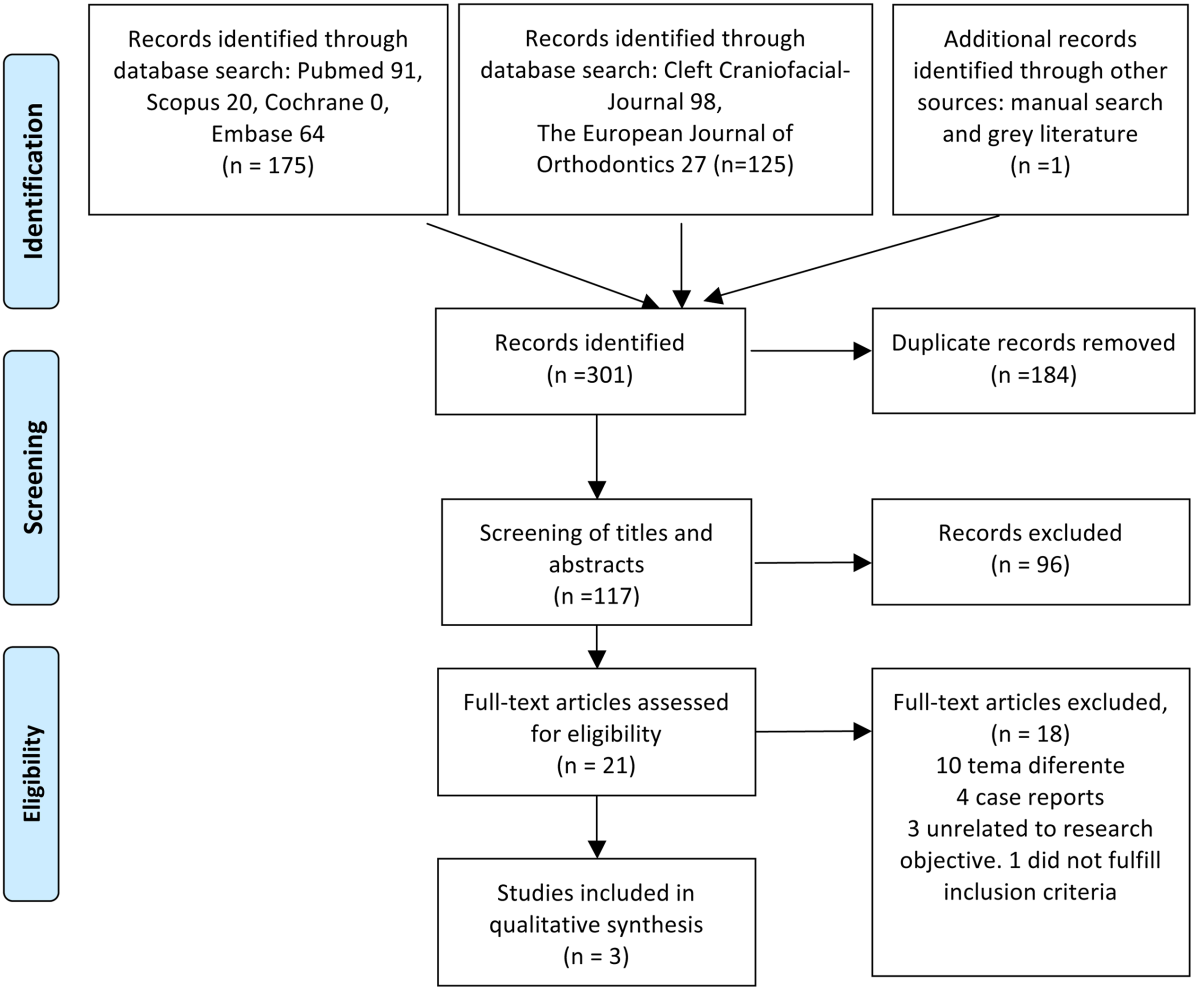
Flow-chart of the selection of studies for the systematic review of predictive validity of the GOSLON Yardstick index. *From*: Moher D, Liberati A, Tetzlaff J, Altman DG, The PRISMA Group (2009). *P*referred *R*eporting *I*tems for Systematic Reviews and *M*eta-*A*nalyses: The PRISMA Statement. PLoS Med 6(7): e1000097. doi: 10.1371/journal.pmed1000097 **For more information, visit** www.prisma-statement.org.

The electronic database search obtained 20 articles in Scopus, 91 articles in Pubmed, 64 articles in Embase and none in Cochrane, a total of 175 articles. A search in the databases of The Cleft Palate-Craniofacial Journal and The European Journal of Orthodontics obtained 98 and 27 articles respectively, totaling 125. Searches in grey literature databases did not locate any more articles. One further reference was added as a result of the manual search. This made a total of 301 articles, of which 184 were duplicates, leaving a total of 117. After reading the titles and abstracts, a further 91 articles were excluded as they did not meet the review’s research objectives, leaving a total of 21 articles. After a complete and detailed reading of the complete manuscripts, only three fulfilled the review’s inclusion criteria. The PRISMA flow diagram (Fig 1) provides an overview of the selection process.

### Study characteristics

The three articles analyzed in the review were longitudinal clinical studies and all presented high methodological quality according to assessment by QUADAS [22] (Table 2).

**Table 2:** Quality of articles evaluated according to QUADAS criteria.

Item		Jones et al., 2016	Suzuki et al., 2014	Sinko et al., 2008
1.	Was the spectrum of patients representative of the patients who will receive the test in practice?	Yes	Yes	Yes
2.	Were selection criteria clearly described?	Yes	Yes	Unclear
3.	Is the reference standard likely to correctly classify the target condition?	Yes	Yes	Yes
4.	Is the time period between reference standard and index test short enough to be reasonably sure that the target condition did not change between the two tests?	Yes	Yes	Yes
5.	Did the whole sample or a random selection of the sample, receive verification using a reference standard of diagnosis?	Yes	Yes	Unclear
6.	Did patients receive the same reference standard regardless of the index test result?	Yes	Unclear	Yes
7.	Was the reference standard independent of the index test (i.e. the index test did not form part of the reference standard)?	Unclear	Yes	Unclear
8.	Was the execution of the index test described in sufficient detail to permit replication of the test?	Yes	Yes	Yes
9.	Was the execution of the reference standard described in sufficient detail to permit its replication?	Yes	Yes	Yes
10.	Were the index test results interpreted without knowledge of the results of the reference standard?	Yes	Yes	Yes
11.	Were the references standard results interpreted without knowledge of the results of the index test?	Yes	Yes	Unclear
12.	Were the same clinical data available when test results were interpreted as would be available when the test is used in practice?	Unclear	Yes	No
13.	Were uninterpretable/intermediate test results reported?	Yes	Yes	Yes
14.	Were withdrawals from the study explained?	Unclear	No	No

All three evaluated the predictive validity of the index Yardstick. But only one, Jones *et al*. [23] compared the GOSLON index with two other indices used to assess UCLP patients: the index for 5-year-old children and the MHB.

The patients born with UCLP in all three studies [23–25] presented characteristics that did not include syndromes or other congenital deformities. Only one study used lateral teleradiography [26], taken at the age of 5 years (85 patients), at 10 years (76 patients) and lateral cephalograms performed at 15 years (54 patients). Across the three studies, the sample sizes varied from 34 patients [23] to 85 patients [26]. All samples contained more men than women. The average age of the patients varied between 5 years [23] and 18 years, 2 months [26].

All the studies expressed the same objective: to analyze the predictive validity of the GOSLON Yardstick index. However, there were a number of differences between the three: Sinko *et al*. [24] had a secondary objective, which was to compare growth in patients born with UCLP treated according to the “Vienna concept” [27] with others treated in the course of the Eurocleft project [13,28,29], a Europe-wide intercenter comparison study) using the GOSLON Yardstick index; Jones *et al*. [23] compared different indices applied to children born with UCLP in order to determine which index was the easiest to use, the most reliable, and the showed the greatest validity and Suzuki *et al*. [26] had a single aim of evaluating maxillofacial growth in patients born with UCLP using the GOSLON Yardstick index.

The methods used by the authors were very similar, as all the studies used plaster dental models of patients born with UCLP, classifying them by means of the GOSLON Yardstick index.

All were longitudinal studies but only one was prospective [26]. In the retrospective studies [23,24], UCLP diagnosis was confirmed in clinical notes but in Sinko *et al*. [24] study an exact diagnosis was established from pre-operative photographs, making study models in centric occlusion. Jones *et al*. [23] relied on clinical histories to obtain the relevant information.

Regarding follow-up periods, Sinko *et al*. [24] evaluated 55 patients at age 17 years out of 123 subjects at the start of the study (mean age 9.2 years); in the prospective longitudinal study by Suzuki *et al*. [26], the study sample of 85 subjects was derived from an earlier study [25] of 136 subjects, only analyzing those subjects with dental models and lateral cephalograms.

All three studies evaluated intra- and inter-examiner reproducibility, obtaining values between 0.41 and 0.95.

### Qualitative synthesis

The different studies found different levels of predictive validity for the GOSLON Yardstick index ranging from 42.4% to 64.7%.

Sinko *et al*. [24] found that 60% of patients maintained the same categorization, 12.7% presented an increase, and 27.3% presented a decrease GOSLON Yardstick scores.

Jones *et al*. [23] comparing GOSLON index scores given over 10 years, found that 64.7% of patients remained in the same category, 17.65% improved in category, and 17.65% worsened in category.

Suzuki *et al*. [26] found that 42.4% of patients did not present changes in category from 5 to 10 years of age, 35.3% showed category improvement, and 22.3% showed a deterioration in growth pattern (Table 3).

**Table 3:** Table detailing the studies selected for analysis and qualitative synthesis.

Author (year) Study type	N (Losses) Men % (n) Women % (n) Mean age	Inclusion and exclusion criteria	Follow-up time	Predictability/ Reproducibility	Conclusions
**Suzuki et al. (2014)** Longitudinal-prospective	136 (51), % M (45), % W (40)	In: Presence of lateral telerradiographs taken at age 15. Ex: Patients with syndromes, patients who had already received upper lateral expansion	**T0:** (61.6 months). **T1:** (10 years and 4 months, SD = 24 months). **T2:** (18 years and 2 months, SD = 40 months	**Improvement**: 30/85(35.3%). **Deterioration**: 19/85 (22.3%). **No change:** 36/85 (42.4%). **IAER:** 0.809–0.832*. **IEER:** 0.665–0.751*.	Boys (14) showed more deterioration than girls (5) (p = 0.056). The GOSLON Yardstick index might not reflect restriction of maxillary growth caused by plastic surgery to repair the lip and palate. In order to predict maxillofacial function of UCLP patients, orthodontists should study the influences of genetics and maxillofacial pubertal growth. Five angles, SNP, SNB, AB plane, facial plane, and facial convexity angle at T1 showed a significant positive correlation, while four angles, AN-B, GZN, FH a SGN, and ramus inclination showed a negative correlation with GOSLON index at T1.
**Sinko et al. (2008)** Longitudinal retrospective	123(68), % M (-), % W (-), 123 subjects (9.2 years)(6–12.5 years), 55 subjects (17 years) (12.5–25 years)	In: Patients with UCLP. Ex: Dental models without bite register	**T0:** 9.2 years (6 and 12.5 years). **T1**: 17 years (12.5 and 25 years)	**Same category**: 33 (60%), **Improvement:** 7 (12.7%), **Deterioration**: (27.3%), **1 category**: 12 (21.8%), **More than 1 category**: 3 (5.5%), **IAER:** 0.66–0.89*, **IEER:** 0.49–0.91*	For GOSLON ratings, 60% of patients maintained the same category and 12.7% increased a category, which could be due to orthodontic treatment of patients' permanent occlusion with fixed apparatus.- Deterioration in 27.3% of cases could be due to inadequate surgical or orthodontic treatment, or unfavorable growth pattern. When the GOSLON index is used by certified evaluators, the system is effective for comparing results of different procedures and for comparing centers.
**Jones et al. (2016)**, Longitudinal retrospective	34 models of patients (-), % M (23), % W (11), 5 years and 3 months, 9 years and 11 months, 18 years and 2 months	In: Patients with UCLP, patients without syndromes, study models available at 5 years and 10 years, final study models of final orthodontic treatment at 15–20 years available	**T0:** 5 years, **T1**: 10 years, **T2**: 20 years	GOLSON results compared at 20 years. **No change:** 64.7%. **Improvemen:** 17.65%. **Deterioratio:** 17.65%. **IAER**: 0.52–0.95*. **IEER**: 0.41–0.70*.	The reality is that the outcome of initial primary surgery is distorted by later surgical and orthodontic treatment, and by the patient's inherent growth pattern. Predictive validity findings were disappointing. The fact that only half of the models remained in the same category emphasized the difficulty of accurately predicting the final outcome and the need for future orthognathic surgery at such a young age.

N = sample size, M = boy, W = girl, In = inclusion criteria, Ex = exclusion criteria, T0 = first time, T1: second time, T2: third time, IAER = intra-examiner reproducibility, IEER = inter-examiner reproducibility.

* = Kappa Value.

## Discussion

According to the literature, the GOSLON Yardstick index is the most widely used system for assessing dental arch relationships in children born with UCLP. It was first introduced in 1987, and so has been available for longer than other methods cited in the literature [30]. It is considered easy to use, and a simple method for assessing the severity of malocclusion [31]. Nevertheless, several authors have claimed that it suffers certain deficiencies including the subjective element involved in assessment and a lack of versatility, as it can only be applied to cases unilateral cleft lip and palate in children with late mixed dentition or early permanent dentition (around the age of 10 years). Furthermore, the introduction of newer systems has led to doubts as to its adequacy [7,8,32–34].

This systematic literature review has highlighted a major lack of longitudinal studies in this area, which makes it impossible to compare our findings with others. When the review’s inclusion criteria had been applied, only three articles remained for analysis. Many works published as case series or case reports were not included, but these would provide valuable information for future studies with higher levels of evidence [35].

According to the three studies analyzed, it can be affirmed that the GOSLON Yardstick index is not capable of predicting growth patterns in patients born with UCLP. In the study by Jones *et al*. [23], the GOSLON index failed to predict case evolution correctly in a third of the patients. Meanwhile, Suzuki *et al*. [26] concluded that growth in UCLP patients is not predictable as it depends on a range of factors such as maxillofacial function, pubertal growth, and genetics. Sinko *et al*. [36] reached similar conclusions, attributing the poor results obtained to bad surgical treatment, unfavorable growth patterns, or failure to perform adequate orthodontic treatments compared with other similar systems.

Of the three studies analyzed, only Suzuki *et al*. [26] analyzed the influence of gender on the GOSLON Yardstick’s index predictive validity, finding that boys showed worse deterioration in scores (31.1%) than girls (12.5%).

The predictive validity of the GOSLON Yardstick index observed in the present review cannot be compared other similar systems due to the lack of longitudinal studies, as only one other longitudinal study [23] compared the GOSLON Yardstick index, the index for 5-year-old children, and the MHB. The predictability at 10 years was poor for all three systems, but the GOSLON index showed greater deterioration than the other two.

Children born with UCLP begin treatment at birth passing through different phases according to their age, determined both surgically and orthodontically by the type of cleft, medical antecedents and whether the malocclusion will permit dentoalveolar compensation or not. Furthermore, treatment will respond to the individual patient’s esthetic and psychosocial requirements and demands. All the studies assessed maxillofacial growth but none looked into patient quality of life, an important aspect for consideration before the patient undergoes orthognathic surgery. Only Sinko *et al*. [36] analyzed patients born with UCLP perceptions of facial esthetics, observing that 44.3% of patients sought complementary esthetic treatments (nose and upper lip correction) in addition to orthognathic surgery.

The main limitation of the present review was the small number of studies located, a finding that provides a partial answer to the review’s research question, in the sense that the predictive validity of the GOLSON Yardstick index has not been investigated sufficiently. In order to limit any publication bias, the search was conducted in four databases, complemented by a search among grey literature and a manual search. Nevertheless, we cannot discount the possibility that there may exist some other study, or studies, that the search failed to locate or that more recently published works might modify the present findings. Nevertheless, on the basis of the present findings, it may be affirmed that there is a lack of evidence in the literature affirning the predictive valitiy of the GOLSON Yardstick.

## Supporting information

S1 Table PRISMA 2009 checklist. (DOC)
